# Predictive Factors for Sentinel Lymph Node Positivity in Melanoma Patients—The Role of Liquid Biopsy, MicroRNA and Gene Expression Profile Panels

**DOI:** 10.3390/cancers17081281

**Published:** 2025-04-10

**Authors:** Federico Venturi, Elisabetta Magnaterra, Biagio Scotti, Manuela Ferracin, Emi Dika

**Affiliations:** 1 Oncologic Dermatology Unit, IRCCS Azienda Ospedaliero-Universitaria di Bologna, 40138 Bologna, Italy; elisabetta.magnaterra@unibo.it (E.M.); biagio.scotti@studio.unibo.it (B.S.); emi.dika3@unibo.it (E.D.); 2 Department of Medical and Surgical Sciences (DIMEC), Alma Mater Studiorum University of Bologna, 40126 Bologna, Italy; manuela.ferracin@unibo.it; 3 IRCCS Azienda Ospedaliero-Universitaria di Bologna, 40138 Bologna, Italy

**Keywords:** sentinel node biopsy, melanoma, predictive factors, liquid biopsy, mirna, GEP

## Abstract

The identification of predictive factors for sentinel lymph node (SLN) positivity in melanoma patients is crucial for accurate staging, prognosis, and personalized therapeutic decisions. This review synthesizes recent advancements in molecular and clinicopathological predictors, with a particular focus on liquid biopsy and gene expression profiling (GEP) tools. Emerging evidence highlights the significant role of miRNAs in melanoma progression, metastatic potential, and lymphatic spread. Clinicopathological factors such as Breslow thickness, ulceration, and mitotic rate remain critical, while GEP provides additional precision by uncovering tumor-specific molecular pathways. By integrating these tools, clinicians can improve risk stratification, reduce unnecessary procedures, and personalize management strategies.

## Simple Summary

Identifying predictive factors for sentinel lymph node (SLN) positivity in melanoma is essential for staging, prognosis, and personalized treatment. This review highlights the role of molecular and clinicopathological predictors, focusing on liquid biopsy, microRNAs and gene expression profiling (GEP). While not yet a routine clinical tool for SLN prediction, ongoing advancements and validation efforts are likely to expand their role in personalized melanoma management. Integrating these tools can improve patient risk stratification, reduce unnecessary procedures, and personalize patient management regimens.

## 1. Introduction

Melanoma incidence is rapidly increasing worldwide, and it represents one of the most frequent tumors affecting young people [1,2,3]. Despite several noninvasive diagnostic tools having recently been integrated into the diagnostic process, leading to earlier detection of thin melanomas in recent years, the incidence of advanced tumors is still a matter of concern [4,5,6]. Furthermore, melanoma mortality rates have generally remained stable. In this scenario, sentinel lymph node biopsy (SLNB) is critical in melanoma management, offering insights into disease staging and guiding therapeutic interventions [7,8,9,10,11]. SLNB is a powerful tool for staging melanoma and predicting patient outcomes. The procedure enables accurate staging as per the American Joint Committee on Cancer (AJCC) melanoma staging system, distinguishing Stage III disease (regional node involvement) from Stage I/II (localized disease) [12]. The presence of melanoma cells in the sentinel node is associated with a significantly worse prognosis. SLNB positivity strictly correlates with a higher risk of recurrence and melanoma-specific mortality [13,14]. Furthermore, it indicates the need for adjuvant therapies with immune checkpoint inhibitors or targeted therapies in BRAF-mutant melanoma [15,16,17]. However, the SLNB procedure is not without controversy or complications, and its associated morbidity emphasizes the growing need for non-invasive predictive biomarkers that may be investigated [18,19]. Traditional clinicopathological factors, while effective, have limitations in sensitivity and specificity. In recent years, several predictive factors, including tumor-derived materials such as circulating tumor DNA (ctDNA) and circulating tumor cells (CTCs), microRNAs (miRNAs), and gene expression profiling (GEP) panels, alone or combined with clinicopathological parameters, have been explored to refine risk stratification for sentinel lymph node (SLN) positivity [20,21,22,23]. Our review highlights them and analyzes their potential clinical implications.

## 2. Methods

The present review was conducted and reported using validated search strategies of the following databases:

- PUBMED
- Ovid MEDLINE

The following keywords and/or MESH terms were used: “cutaneous melanoma” OR “melanoma” AND “liquid biopsy” AND “circulating tumor DNA” OR “ctDNA” AND “circulating tumor cells” OR “CTCs” AND “extracellular vesicles” OR “EVs” AND “microRNA” OR “miRNA” AND “prognostic gene signature” OR “prognostic genetic signature” OR “gene expression profile” OR “gene expression profiling” OR “GEP” AND “sentinel lymph node”. The search was limited to studies published prior to January 2025, and the references of included studies were also reviewed to identify additional relevant studies.

## 3. Liquid Biopsy

Liquid biopsy is a minimally invasive tool that assesses tumor-derived materials circulating in the blood, including circulating tumor DNA (ctDNA), circulating tumor cells (CTCs), extracellular vesicles (EVs), and microRNAs (miRNAs) [24,25,26,27,28,29] (Table 1).

### 3.1. Circulating Tumor DNA (ctDNA)

CtDNA originates from fragmented tumor-derived genetic material found in the bloodstream. It may result from apoptotic and necrotic tumor cells and indicates tumor load as well as the likelihood of metastatic spread to lymph nodes [30]. Specifically, quantitative levels of ctDNA correlate with tumor size and depth, which are important drivers of SLN metastasis, and detectable mutations in ctDNA (BRAF, NRAS, CDKN2A, and TERT promoter mutations) are linked with a higher risk of SLN positivity [31,32]. Patients with detectable ctDNA prior to SLNB are more likely to have SLN involvement, especially in advanced melanomas. Furthermore, their levels fall following surgical excision, suggesting that they might be used in post-operative surveillance. In this view, the incorporation of ctDNA analysis into SLNB selection criteria may serve as an addition to established risk factors (Breslow thickness, ulceration, and mitotic rate) for better patient stratification.

### 3.2. Circulating Tumor Cells (CTCs)

CTCs originate from the primary tumor and enter systemic circulation either passively through disrupted vasculature or actively via epithelial–mesenchymal transition (EMT). Their presence in peripheral blood suggests a higher metastatic potential. CTCs correlate with systemic dissemination of melanoma, including SLN metastases as higher CTC counts have been associated with increased tumor burden, deeper Breslow thickness, and ulceration (factors linked to SLN positivity) [33,34,35].

### 3.3. Extracellular Vesicles (EVs)

Many cell types, including melanoma cells, release EVs, which are membrane-bound vesicles at the nanoscale. Apoptotic bodies (>1000 nm) are released following programmed cell death, microvesicles (100–1000 nm) are produced by direct plasma membrane budding, and exosomes (30–150 nm) are produced by the endosomal pathway and are involved in cell communication. Melanoma-derived EVs have been implicated in metastasis by facilitating immune evasion, promoting angiogenesis, and conditioning the pre-metastatic niche. EV-based liquid biopsies hold promise for predicting SLN positivity with minimal invasiveness. Several studies have shown that EV-derived biomarkers correlate with disease progression and SLN involvement [36,37,38,39].

## 4. Prognostic MicroRNAs in Melanoma

miRNAs are small, non-coding RNAs that control post-transcriptional expression of genes. Tumor development, angiogenesis, immune evasion, and metastasis have all been connected to their dysregulation in melanoma [40,41]. They play an important role by targeting immune checkpoint inhibitors in the tumor microenvironment and their expression profile can be used as a potential biomarker to predict the response and clinical outcomes in cancer immunotherapy and chemotherapy [42]. Moreover, miRNAs are implicated in the BRAF signaling pathway modulating the response to target therapies [43].

Important microRNAs linked to SLN positivity include miR-21, which is overexpressed in metastatic melanoma and promotes immunological suppression and lymphangiogenesis. An oncogenic microRNA (oncomiR), miR-21, targets tumor suppressor genes such as TIMP3, PDCD4, and PTEN. Through the modulation of pathways such as TGF-β and PI3K/AKT, it facilitates invasion, migration, proliferation, and survival. miR-21 promotes the breakdown of the extracellular matrix and increases the spread of metastases by inhibiting tumor suppressors [23]. miR-205 suppresses epithelial–mesenchymal transition (EMT) by targeting ZEB1 and ZEB2, inhibits invasion and metastasis, and acts as a tumor suppressor in cutaneous melanoma. Its downregulation correlates with increased SLN positivity, possibly due to its role in epithelial–mesenchymal transition (EMT) [44]. miR-125b regulates differentiation, proliferation, and apoptosis by negatively modulating NCAM (neural cell adhesion molecule) expression. It also inhibits EMT and angiogenesis and is inversely associated with metastatic potential; low levels are linked to SLN involvement [45]. miR-155 promotes immune evasion by inhibiting immune response regulators, such as SOCS1 (Suppressor of Cytokine Signaling 1). It contributes to chronic inflammation and supports melanoma progression through the NF-κB and JAK/STAT pathways. Poor immune surveillance is associated with its overexpression, which enables melanoma cells to avoid detection and spreading to lymph nodes [46,47]. The transcription factor HOXD10, which inhibits cell invasion and migration, is the target of miR-10b. RhoC, a GTPase that promotes cytoskeletal reorganization, cell movement, and the potential for metastasis, is activated by its overexpression [48]. In SLN-positive melanoma, elevated miR-10b levels are regularly seen, suggesting that it plays a part in starting the metastatic cascade [28,49,50]. miR-203 suppresses tumors by blocking EMT-related genes like ZEB2 and Snail and pathways like Wnt/β-catenin [51]. Additionally, it encourages melanoma cell differentiation and suppresses proliferation. Downregulation of miR-203 enhances melanoma cell invasiveness and is a biomarker for lymph node metastasis [52]. miR-146a regulates immune responses by targeting TRAF6 and IRAK1, key mediators of the NF-κB signaling pathway. It also modulates inflammation and reduces tumor-promoting immune cell infiltration. Decreased miR-146a expression promotes immune suppression in the tumor microenvironment, increasing the likelihood of SLN metastasis [23]. miR-214 is involved in angiogenesis and cellular stress responses. Elevated miR-214 levels are linked to resistance to apoptosis and an increased likelihood of melanoma spread to lymph nodes [53]. miR-34a, a tumor suppressor regulated by p53, inhibits cell-cycle progression and promotes apoptosis. It directly targets BCL2, CDK4/6, and EMT-related genes like Snail [54]. Low expression of miR-34a in SLN-positive melanoma is associated with reduced apoptosis and increased metastatic potential. miR-182 promotes tumor growth and invasion by the potential target of APC and tumor suppressors like MITF. Overexpression of miR-182 is associated with aggressive tumor phenotypes and a higher probability of SLN involvement [55,56]. Results are fully listed in Table 2.

## 5. Gene Expression Profiling (GEP)

GEP panels assess how particular gene sets are expressed in melanoma tumor cells [30]. In order to give predictive information beyond conventional clinicopathological markers, such as Breslow thickness, ulceration, and sentinel lymph node status, these gene expression patterns are examined [57,58,59]. GEP panels are commonly used to stratify patients into low-risk or high-risk categories for recurrence or metastasis. By identifying high-risk patients, GEP panels assist in guiding adjuvant therapy. Patients with high-risk GEP results may benefit from adjuvant immune checkpoint inhibitors (nivolumab and pembrolizumab) or targeted therapies (BRAF/MEK inhibitors in BRAF-mutant melanoma). Low-risk patients may be able to avoid overtreatment while high-risk patients may need more frequent imaging, clinical examinations, or SLNB [60,61]. Furthermore, in the staging of melanoma, GEP panels have been investigated as a supplement to SLNB. GEP can enhance risk stratification for melanomas with an intermediate thickness (1.0–4.0 mm) by supplementing SLNB data [62]. Pathways relevant to SLN positivity include immune evasion (downregulation of antigen presentation pathways), lymphangiogenesis (upregulation of VEGF-C and VEGF-D signaling), cell cycle, and proliferation (dysregulation of CDKN2A, BRAF, and NRAS mutations). In patients who are SLNB-negative, a high-risk GEP result may warrant closer follow-up and consideration for adjuvant therapy [63,64,65]. GEP tests, including 31-GEP, CP-GEP (Merlin^TM^), and MelaGenix, improve melanoma risk stratification (Table 3).

### 5.1. The 31-GEP Test

With the aim of categorizing melanoma patients into high and low-risk prognostic categories irrespective of pathologic characteristics, the 31-GEP test (DecisionDx-Melanoma, Castle Biosciences, Friendswood, TX, USA) was established in 2015. In order to determine metastatic risk, it evaluates the variations in gene expression between primary and metastatic melanomas and assigns a score between 0 and 1 [66,67]. In order to improve risk assessment, patients are further classified into Class 1 (low risk) or Class 2 (high risk), with further subclassifications (A and B). The 31-GEP test has been criticized for its inability to be compared to conventional clinicopathologic criteria and its application in sentinel lymph node biopsy (SLNB) decision-making. To address this, the i31-SLNB algorithm was introduced, integrating clinicopathologic variables with the 31-GEP score to refine SLN positivity risk assessment [68]. The i31-ROR model, an additional improvement, calculates 5-year distant metastasis-free survival (DMFS), recurrence-free survival (RFS), and melanoma-specific survival (MSS) based on SLNB results.

### 5.2. The Clinicopathologic and Gene Expression Profile Model (CP-GEP, Merlin^TM^)

Similarly to i31-SLNB, the CP-GEP model, Merlin^TM^ (SkylineDx, Rotterdam, The Netherlands) combines clinicopathologic criteria with a distinct gene signature to identify patients with a <5% probability of SLN metastasis [69]. Developed from melanoma patients with nodal metastases, the model incorporates eight genes linked to epithelial–mesenchymal transition and clinicopathologic variables (e.g., age, Breslow depth). Studies report a negative predictive value (NPV) of 96% for nodal metastasis and an area under the curve (AUC) of 0.82, with a potential 42% reduction in unnecessary SLNB procedures [70]. Validation studies confirm its effectiveness in predicting SLN positivity and melanoma recurrence risk. The prediction accuracy of CP-GEP is higher than that of the MSKCC nomogram. Its function in risk stratification is further supported by RFS analyses, which show noticeably inferior results for high-risk individuals.

### 5.3. The 8-GEP Test (MelaGenix)

MelaGenix (Neracare, Frankfurt, Germany), which is sold in Europe, uses qRT-PCR to evaluate three reference genes and eight prognostic genes to improve melanoma prognostication. It produces a continuous score that has a strong correlation with 5-year MSS and stratifies patients into low-risk or high-risk groups [71]. Clinical validation studies show that it has a complementary role in AJCC staging, especially in helping patients with Stage II melanoma make decisions about adjuvant therapy.

## 6. Clinicopathological (CP) Features

While molecular tools are promising tools for improving risk stratification, clinicopathological features remain essential in SLN positivity prediction [72,73]. Among them, traditional markers include Breslow thickness, which is the strongest predictor, particularly for melanoma with a depth of ≥0.8 mm, ulceration, associated with higher SLN positivity and poorer outcomes, and mitotic rate, which reflects tumor aggressiveness and correlates with SLN involvement [74,75,76,77]. According to recent research, prediction accuracy is increased when miRNA profiles and GEP are combined with clinicopathological variables. For example, ulceration status and a high-risk miRNA profile together offer better risk classification [23].

## 7. Conclusions

Predicting SLN positivity in melanoma patients is still being discussed and requires the integration of molecular and clinicopathological data. Liquid biopsy represents a promising non-invasive tool for predicting SLN positivity in melanoma patients. ctDNA, CTCs, EVs, and miRNAs offer valuable insights into tumor burden, metastatic potential, and SLN involvement. While not yet a routine clinical tool for SLN prediction, ongoing advancements and validation efforts are likely to expand its role in personalized melanoma management. GEP procedures provide a molecular dimension to conventional clinicopathological predictors. Integrating these techniques allows clinicians to improve risk classification, avoid unnecessary procedures, and personalize management regimens. Further validation in prospective clinical trials, as well as the creation of standardized methods, is essential for guaranteeing the progressive integration of such prognostic biomarkers in our clinical practice.

## Figures and Tables

**Table 1:** Liquid biopsy as a predictive factor for sentinel lymph node (SLN) positivity in melanoma patients.

Liquid Biopsy Component	Description	Predictive Role for SLN Positivity	Advantages	Limitations	References
Circulating Tumor DNA (ctDNA)	DNA fragments shed by tumor cells into the bloodstream.	Contains tumor-specific mutations (e.g., BRAF, NRAS).	Correlates with tumor burden and micrometastases. Mutations like BRAF and NRAS are associated with SLN involvement.	Limited sensitivity in early-stage melanoma. False negatives in low tumor burden. High cost of testing.	[30,31,32]
Circulating Tumor Cells (CTCs)	Intact tumor cells released into the bloodstream.	Detected using immunomagnetic enrichment or cytology.	Higher CTC counts are linked to SLN positivity. Reflects metastatic potential and tumor aggressiveness.	Rare in early-stage melanoma. Limited detection sensitivity. Requires advanced technology.	[33,34,35]
Extracellular Vesicles (EVs)	Tumor-derived vesicles (e.g., exosomes) carrying DNA, RNA and and proteins.	Biomarkers include S100B and TYRP1.	EV content reflects melanoma progression. Presence of melanoma-specific markers linked to SLN involvement.	Limited clinical validation. Technologically complex assays. High cost.	[36,37,38,39]
Circulating MicroRNAs (miRNAs)	Small, non-coding RNA molecules involved in gene regulation.	Specific miRNAs (e.g., MEL12 signature) are dysregulated in melanoma.	miRNA profiles correlate with SLN positivity. Can identify high-risk patients.	Limited sensitivity and specificity. Requires further standardization and validation.	[22]

**Table 2:** MicroRNAs associated with sentinel lymph node (SLN) positivity in melanoma.

MicroRNA	Specific Function	Correlation with SLN Positivity	Notes	References
miR-21	Promotes tumor progression by targeting tumor suppressors (PTEN and PDCD4).	Overexpression is significantly associated with SLN positivity	Frequently upregulated in melanoma.	[23]
miR-205	Suppresses EMT by targeting ZEB1 and ZEB2 and inhibits invasion and metastasis.	Downregulated in SLN-positive melanoma, contributing to increased EMT.	Acts as a tumor suppressor in melanoma.	[44]
miR-125b	Controls differentiation and proliferation by targeting NCAM.	Low levels are predictive of SLN metastasis.	Prognostic marker for melanoma.	[45]
miR-155	Enhances inflammation and immune evasion in the tumor microenvironment.	Correlated with increased risk of SLN metastasis.	Marker of poor prognosis.	[46,47]
miR-10b	Facilitates cell migration and invasion by targeting HOXD10.	Overexpressed in SLN-positive melanoma cases.	Associated with metastatic potential.	[28,48,49,50]
miR-203	Regulates cell proliferation and epithelial-to-mesenchymal transition (EMT).	Downregulated in SLN-positive melanomas.	Loss may promote metastasis.	[51,52]
miR-146a	Regulates immune response and NF-κB signaling.	Reduced expression linked to SLN positivity and immune suppression.	Acts as a tumor suppressor.	[23]
miR-214	Involved in cell survival and resistance to apoptosis.	Elevated levels associated with SLN positivity and melanoma progression.	Key in chemoresistance mechanisms.	[53]
miR-34a	Tumor suppressor regulating cell cycle and apoptosis via p53.	Reduced expression observed in SLN-positive patients.	Promotes sensitivity to therapies.	[54]
miR-182	Enhances tumor growth, invasion, and angiogenesis.	Overexpression linked to SLN metastasis and aggressive tumor phenotype.	Frequently altered in late-stage melanoma.	[55,56]

**Table 3:** Gene expression profiling tests for melanoma prognosis.

Test	Purpose	Risk Classification	Key Features	References
31-GEP (DecisionDx-Melanoma)	Stratifies melanoma patients into high/low metastatic risk.	Class 1A (lowest risk) to Class 2B (highest risk).	Uses gene expression differences between primary and metastatic melanomas. Integrated with i31-SLNB for SLN positivity risk. - i31-ROR model estimates 5-year RFS, DMFS, and MSS.	[66,67,68]
CP-GEP (Merlin™)	Identifies patients with <5% risk of SLN metastasis.	Low risk vs. high risk of nodal metastasis.	Uses eight genes + clinicopathologic factors (age and Breslow depth). - 96% NPV for nodal metastasis. - 42% SLNB reduction rate. - Higher predictive accuracy than MSKCC nomogram.	[69,70]
8-GEP (MelaGenix)	Provides prognostic information for melanoma-specific survival (MSS).	Continuous score (−0.84 to 3.55) Low-risk (<1.3) vs. High-risk (≥1.3).	- Uses qRT-PCR for 8 prognostic genes + 3 reference genes. - Strong correlation with 5-year MSS (AUC = 0.91 with AJCC staging). - Designed to aid adjuvant therapy decisions.	[71]

## Data Availability

No new data were created or analyzed in this study. Data sharing is not applicable to this article.
